# A New Correlation Measure for Belief Functions and Their Application in Data Fusion

**DOI:** 10.3390/e25060925

**Published:** 2023-06-12

**Authors:** Zhuo Zhang, Hongfei Wang, Jianting Zhang, Wen Jiang

**Affiliations:** 1 School of Electronics and Information, Northwestern Polytechnical University, Xi’an 710072, China; zhangzhuonwpu@126.com (Z.Z.); wanghongfeinwpu@126.com (H.W.); 2 No. 91977 Unit of People’s Liberation Army of China, Beijing 100036, China; changjianting@hotmail.com; 3 National Engineering Laboratory for Integrated Aero-Space-Ground-Ocean Big Data Application Technology, Xi’an 710072, China; 4 Peng Cheng Laboratory, Shenzhen 518055, China

**Keywords:** Dempster–Shafer theory, belief correlation measure, uncertainty, information fusion, multi-source data

## Abstract

Measuring the correlation between belief functions is an important issue in Dempster–Shafer theory. From the perspective of uncertainty, analyzing the correlation may provide a more comprehensive reference for uncertain information processing. However, existing studies about correlation have not combined it with uncertainty. In order to address the problem, this paper proposes a new correlation measure based on belief entropy and relative entropy, named a belief correlation measure. This measure takes into account the influence of information uncertainty on their relevance, which can provide a more comprehensive measure for quantifying the correlation between belief functions. Meanwhile, the belief correlation measure has the mathematical properties of probabilistic consistency, non-negativity, non-degeneracy, boundedness, orthogonality, and symmetry. Furthermore, based on the belief correlation measure, an information fusion method is proposed. It introduces the objective weight and subjective weight to assess the credibility and usability of belief functions, thus providing a more comprehensive measurement for each piece of evidence. Numerical examples and application cases in multi-source data fusion demonstrate that the proposed method is effective.

## 1. Introduction

The uncertainty of information is mainly manifested as vagueness, unknown, inaccuracy, etc. [1,2]. At present, related methods of uncertain information processing have been widely applied to decision making [3,4], image classification [5,6], and many other fields [7,8,9]. For example, in target recognition tasks, since the uncertainty is caused by the dynamicity of the environment in which the target is located, it is difficult to make an accurate recognition [10]. Meanwhile, due to the uncertainty caused by the unreliability of sensors, such as sensor failures and noise interference, the data collected by some sensors may be incorrect, leading to the possibility of making wrong decisions [11]. Similarly, in practical application of other fields, information uncertainties are also inevitable [12,13]. How to deal with these uncertainties or achieve information fusion is an important issue in information processing [14,15]. To resolve this problem, several theories have been developed, including Dempster–Shafer theory (D-S theory) [16,17,18], fuzzy set theory [19,20,21], Z numbers [22,23,24], evidential clustering [25,26,27,28], and so on [29,30,31]. Among them, the D-S theory, also known as belief function theory, is a representative uncertainty reasoning method. It is an extension and evolution of the Bayesian probability theory and satisfies weaker axiomatic conditions [32]. It also provides effective means to model uncertain information and combine pieces of evidence [33]. Therefore, D-S theory has a strong ability to deal with uncertainty.

Although D-S theory has many advantages, in practical application, conflicts between evidence sometimes lead to counter-intuitive fusion results, which seriously affect the reliability of decision-making systems [34,35]. Uncertainty is one of the important reasons for conflicts between evidence [36]. Therefore, in the process of conflict management, the measurement of information uncertainty is an issue worth studying. Inspired by Shannon’s information entropy [37], which solves the problem of information uncertainty measurement in probability theory, Deng [38] proposed a new uncertainty measurement method for belief functions, which is called Deng entropy or belief entropy. Belief entropy can quantify the uncertainty of evidence based on the belief function and has been used in many fields [39,40,41,42].

In D-S theory, uncertainty includes the discord and non-specificity that co-exist in a basic probability assignment (BPA) [43,44]. The measurement of correlation or divergence between evidence can quantify the inconsistency, and information fusion based on correlation measures provides an idea for uncertain information processing [45]. In order to measure conflict and deal with uncertainty from the perspective of evidence correlation, Jiang [46] proposed a correlation coefficient to carry out a reasonable measurement of the correlation between evidence. In addition, the relevance and divergence of information can be transformed into each other. Kullback–Leibler divergence (KLD) [47] is a classical measurement for the difference between probability distributions. On the basis of KLD, Xiao [48] proposed a belief divergence measure for belief functions and Gao [49] developed a generalized divergence measure. In addition, both of them converted divergence measure into correlation measures and then put forward an information fusion method, which achieved good results in uncertain information processing. However, these measurement methods only consider the correlation or difference between basic probability assignments, and ignore the influence of uncertainty itself on the measurement of information correlation.

In order to analyze evidence more comprehensively, this paper proposes a new correlation measurement method, which is named a belief correlation measure. It combines information uncertainty to measure the correlation between belief functions. On the basis of a belief correlation measure and a discriminability measure, an information fusion method for multi-source data is proposed, which can produce more reasonable decision results.

The main contributions made in this work are:

(a) The belief correlation measure based on belief entropy and relative entropy is proposed, which combines uncertainty to measure the correlation between belief functions. It not only provides a more comprehensive correlation analysis, but also has some important mathematical properties such as probabilistic consistency, non-negativity, non-degeneracy, boundedness, orthogonality, and symmetry.

(b) An information fusion method for multi-source data is proposed. It introduces objective weights based on the belief correlation measure and subjective weight based on the discriminability measure. Then, the combinational weight is designed to perform evidence fusion, which can improve the rationality and reliability of decision results.

(c) The effectiveness of the proposed belief correlation measure is illustrated by several numerical examples. Then, two application cases of multi-source data are analyzed to demonstrate the advantage of the proposed information fusion method.

The organization of this paper is as follows. The concept and connotation of related theories are described in Section 2. The proposed belief correlation measure and the mathematical properties are elaborated in Section 3. Several illustrative examples are presented to demonstrate that the belief correlation measure is reasonable in Section 4. Then, an information fusion method for multi-source data is proposed in Section 5. Two specific application cases are described in steps in Section 6, while the comparisons are discussed to verify the effectiveness of the method. At last, the whole work is summarized in Section 7 with an outlook for future work.

## 2. Preliminaries

### 2.1. D-S Theory

D-S theory is a classical uncertainty reasoning theory. It satisfies weaker conditions than probability theory, and can express the uncertainty of information more directly [50].

In D-S theory, assume that Θ is a set consisting of *n* mutually exclusive objects, Θ is named the frame of discernment (FOD):

(1) $$\Theta \;=\;\{\theta_{1},\theta_{2},\ldots ,\theta_{i},\ldots ,\theta_{n}\}$$

The power set of Θ is defined as $2^{\Theta }$:

(2) $$2^{\Theta }=\left\{\phi ,\left\{\theta_{1}\right\},\left\{\theta_{2}\right\},\ldots ,\left\{\theta_{1},\theta_{2}\right\},\ldots ,\Theta \right\}$$

where ϕ represents the empty set. It can be obtained that there are $2^{n}$ elements in $2^{\Theta }$. Each element of $2^{\Theta }$, which is also the subset of Θ, corresponds to a proposition with the possible value.

Suppose there is a proposition *A* belonging to $2^{\Theta }$. The belief function *m*, also known as BPA, maps *A* from $2^{\Theta }$ to [0, 1] and satisfies:

(3) $$\begin{array}{c} \left(i\right)\;m:2^{\Theta }\to \left[0,1\right]\\ \left(ii\right)\;\sum_{A\in 2^{\Theta }}m(A)=1\\ (iii)\;m(\phi )=0 \end{array}$$

where the mass value m(A) indicates the support degree for proposition *A*. If A⊆Θ and m(A)>0, *A* is referred to as a focal element.

Assume belief functions $m_{1}$ and $m_{2}$ are two independent belief functions, Dempster’s combination rule provides an efficient way to aggregate them:

(4) $$m\left(A\right)=\left\{\begin{array}{cc} \frac{1}{1-K}\sum_{B\cap C=A}m_{1}\left(B\right)m_{2}\left(C\right), & A\neq \phi \\ 0, & A=\phi \end{array}\right.$$

where

(5) $$\begin{array}{c} K=\sum_{B\cap C=\phi }m_{1}\left(B\right)m_{2}\left(C\right) \end{array}$$

The significance of combining evidence with the orthogonal sum method is in scaling the conflicting mass *K* proportionally to the fusion results. Dempster’s rule of combination can fuse evidence without prior information, thus enabling efficient processing of uncertain information [51].

### 2.2. Belief Entropy

Shannon entropy solves the problem of quantitative information measurement in probability theory [52]. Inspired by Shannon entropy, Deng [38] proposed belief entropy, which can measure information uncertainty under the theoretical framework of belief function theory.

The belief entropy of BPA *m* is defined as:

(6) $$\begin{array}{cc} E(m) & =-\sum_{A\subseteq \Theta }m\left(A\right)\log_{2}\frac{m(A)}{2^{\left|A\right|}-1}\\ \; & =\sum_{A\subseteq \Theta }m\left(A\right)\log_{2}(2^{\left|A\right|}-1)-\sum_{A\subseteq \Theta }m\left(A\right)\log_{2}m\left(A\right) \end{array}$$

where |A| is the cardinality of set *A*. The separated two parts in belief entropy can quantify non-specificity and discord of a BPA.

If the mass value of a BPA is assigned to the subsets with single elements, then belief entropy is transformed into Shannon entropy:

(7) $$E(m)=-\sum_{A\in \Theta }m\left(A\right)\log_{2}m\left(A\right)$$

The probabilistic consistency of belief entropy makes it have better generalizability. In addition, when further studying the belief entropy and its applications, some limitations, including non-monotonicity, non-additivity, etc., should also be noticed [53].

### 2.3. Relative Entropy of Random Variable

The partial entropy in probability theory, and the relative entropy of a random variable derived from it have been applied in the field of uncertain information recently. The specific definitions are as follows [54]:

Suppose there are two sets of random variables *X* and *Y*, their distributions are:

(8) $$\begin{array}{c} X:\left\{\begin{array}{c} x_{1},\ldots ,x_{K}\\ p_{1},\ldots ,p_{K} \end{array}\right\};\\ Y:\left\{\begin{array}{c} y_{1},\ldots ,y_{K}\\ q_{1},\ldots ,q_{K} \end{array}\right\} \end{array}$$

where $x_{i}\left(i=1,\ldots ,K\right)$ is the random variable in *X*, and $p_{i}$ is the corresponding probability of $x_{i}$. $y_{i}$ is the random variable in *Y*, and $q_{i}$ is the corresponding probability of $y_{i}$. The definition of entropy for variable *X* is:

(9) $$H\left(X\right)=-\sum_{k=1}^{K}p_{i}\log_{2}p_{i}$$

The partial entropy of variable *X* with respect to variable *Y* is:

(10) $$H_{Y}\left(X\right)=-\sum_{k=1}^{K}q_{i}\log_{2}p_{i}$$

The relative entropy of the pair of variables *X* and *Y* is:

(11) $$\begin{array}{cc} H(X;Y) & =H_{Y}\left(X\right)+H_{X}\left(Y\right)\\ \; & =-\sum_{k=1}^{K}q_{i}\log_{2}p_{i}-\sum_{k=1}^{K}p_{i}\log_{2}q_{i} \end{array}$$

## 3. The Proposed Belief Correlation Measure

Belief entropy has increased the study of uncertainty measurements based on the belief function. Relative entropy can describe the correlation between probability distributions. How to combine the uncertainty information contained in the belief function to describe the correlation degree between BPAs is worth studying. Therefore, this paper proposes a new correlation measure on the basis of belief entropy and correlation coefficient, which is named the belief correlation measure. The specific details are described as follows.

### 3.1. Definition of Belief Correlation Measure

Assume there are two BPAs $m_{1}$ and $m_{2}$:

$$\begin{array}{c} m_{1}:\left\{m_{1}\left(A_{1}\right),m_{1}\left(A_{2}\right),\ldots ,m_{1}\left(A_{N}\right)\right\}\\ m_{2}:\left\{m_{2}\left(A_{1}\right),m_{2}\left(A_{2}\right),\ldots ,m_{2}\left(A_{N}\right)\right\} \end{array}$$

The partial belief entropy of $m_{1}$ with respect to $m_{2}$ is defined as:

(12) $$E_{m_{2}}\left(m_{1}\right)=-\sum_{i=1}^{N}m_{2}\left(A_{i}\right)\log_{2}\frac{m_{1}\left(A_{i}\right)}{2^{\left|A_{i}\right|}-1}$$

The relative belief entropy between the belief function $m_{1}$ and $m_{2}$ is the sum of their partial belief entropy, which is defined as:

(13) $$\begin{array}{cc} E(m_{1};m_{2}) & =E_{m_{2}}\left(m_{1}\right)+E_{m_{1}}\left(m_{2}\right)\\ \; & =-\sum_{i=1}^{N}m_{2}\left(A_{i}\right)\log_{2}\frac{m_{1}\left(A_{i}\right)}{2^{\left|A_{i}\right|}-1}-\sum_{i=1}^{N}m_{1}\left(A_{i}\right)\log_{2}\frac{m_{2}\left(A_{i}\right)}{2^{\left|A_{i}\right|}-1} \end{array}$$

The partial belief correlation coefficient of evidence $m_{1},m_{2}$ are defined as follows:

(14) $$\begin{array}{cc} \gamma_{m_{2}}\left(m_{1}\right) & =\frac{E(m_{2})}{E_{m_{2}}\left(m_{1}\right)}\\ \gamma_{m_{1}}\left(m_{2}\right) & =\frac{E(m_{1})}{E_{m_{1}}\left(m_{2}\right)} \end{array}$$

where $E(m_{2})$ is the belief entropy of $m_{2}$ defined in Equation (6). Then, the belief correlation measure of $m_{1}$ and $m_{2}$ is defined as:

(15) $$\begin{array}{c} \gamma \left(m_{1};m_{2}\right)=\frac{E\left(m_{1}\right)+E\left(m_{2}\right)}{E_{m_{2}}\left(m_{1}\right)+E_{m_{1}}\left(m_{2}\right)} \end{array}$$

It is worth noting that in the actual calculation process, when the mass value of $m_{1}\left(A_{i}\right)$ or $m_{2}\left(A_{i}\right)$ is 0, its logarithm tends to infinity, which makes it impossible to calculate. Therefore, this method replaces 0 with a smaller value of $10^{-12}$. It has been proved in Ref. [55], that the setting of this value has no influence on the calculation results.

### 3.2. Properties of Belief Correlation Measure

When analyzing the mathematical properties of correlation measures, it is usually necessary to consider such properties as probabilistic consistency, non-negativity, non-degeneracy, boundedness, orthogonality, symmetry, and triangular inequality [46,48,49,56]. In this section, we prove the properties possessed by the belief correlation measure.

**Property** **1** (Probabilistic consistency)**.** *When two BPAs $m_{1}$ and $m_{2}$ are degenerated as probability distributions, the belief correlation measure of them can be degenerated to the correlation coefficient in probability theory.*

**Proof.**  When the mass value is assigned to single elements, the belief entropy values $E(m_{1})$ and $E(m_{2})$ are reduced to Shannon entropy:

$$\begin{array}{c} E\left(m_{1}\right)=-\sum_{i=1}^{N}m_{1}\left(A_{i}\right)\log_{2}m_{1}\left(A_{i}\right)\\ E\left(m_{2}\right)=-\sum_{i=1}^{N}m_{2}\left(A_{i}\right)\log_{2}m_{2}\left(A_{i}\right) \end{array}$$

The partial belief entropy values $E_{m_{2}}\left(m_{1}\right)$ and $E_{m_{1}}\left(m_{2}\right)$ are reduced to partial entropy:

$$\begin{array}{c} E_{m_{2}}\left(m_{1}\right)=-\sum_{i=1}^{N}m_{2}\left(A_{i}\right)\log_{2}m_{1}\left(A_{i}\right)\\ E_{m_{1}}\left(m_{2}\right)=-\sum_{i=1}^{N}m_{1}\left(A_{i}\right)\log_{2}m_{2}\left(A_{i}\right) \end{array}$$

Then, the belief correlation measure $\gamma (m_{1};m_{2})$ is degenerated to:

$$\begin{array}{c} \begin{array}{cc} \gamma (m_{1};m_{2}) & =\frac{E\left(m_{1}\right)+E\left(m_{2}\right)}{E_{m_{2}}\left(m_{1}\right)+E_{m_{1}}\left(m_{2}\right)}\\ & =\frac{-\sum_{i=1}^{N}m_{1}\left(A_{i}\right)\log_{2}m_{1}\left(A_{i}\right)-\sum_{i=1}^{N}m_{2}\left(A_{i}\right)\log_{2}m_{2}\left(A_{i}\right)}{-\sum_{i=1}^{N}m_{2}\left(A_{i}\right)\log_{2}m_{1}\left(A_{i}\right)-\sum_{i=1}^{N}m_{1}\left(A_{i}\right)\log_{2}m_{2}\left(A_{i}\right)} \end{array} \end{array}$$

It is consistent with the correlation coefficient V(X;Y) in probability theory [55]:

$$\begin{array}{c} \begin{array}{cc} V(X;Y) & =\frac{H(X)+H(Y)}{H_{Y}\left(X\right)+H_{X}\left(Y\right)}\\ & =\frac{-\sum_{i=1}^{N}p_{i}\log_{2}p_{i}-\sum_{i=1}^{N}q_{i}\log_{2}q_{i}}{-\sum_{i=1}^{N}q_{i}\log_{2}p_{i}-\sum_{i=1}^{N}p_{i}\log_{2}q_{i}} \end{array} \end{array}$$

Thus, the probabilistic consistency of the belief correlation measure is proved. □

**Property** **2** (Non-negativity)**.** *$\gamma (m_{1};m_{2})\geq 0$.*

**Proof.**  Suppose *P* and *Q* are probability distributions from [47], we know that the KLD is non-negative, that is:

$$D_{KL}\left(P\|Q\right)=\sum_{i=1}^{k}p_{i}\log_{2}\frac{p_{i}}{q_{i}}=\sum_{i=1}^{k}p_{i}\log_{2}p_{i}-\sum_{i=1}^{k}p_{i}\log_{2}q_{i}\geq 0$$

Through the derivation process:

(16) $$\begin{array}{c} \sum_{i=1}^{k}p_{i}\log_{2}q_{i}\leq \sum_{i=1}^{k}p_{i}\log_{2}p_{i}\leq 0\\ \Rightarrow \sum_{i=1}^{k}p_{i}\log_{2}\frac{q_{i}}{c}\leq \sum_{i=1}^{k}p_{i}\log_{2}\frac{p_{i}}{c}\leq 0 \end{array}$$

where *c* is a constant and c≥1. It can be obtained that:

$$\begin{array}{c} -\sum_{i=1}^{k}p_{i}\log_{2}\frac{q_{i}}{c}\geq \;0\\ -\sum_{i=1}^{k}p_{i}\log_{2}\frac{p_{i}}{c}\geq 0 \end{array}$$

With Equations (6) and (12), it can be obtained that:

$$\begin{array}{c} E\left(m_{1}\right)=-\sum_{i=1}^{N}m_{1}\left(A_{i}\right)\log_{2}\frac{m_{1}\left(A_{i}\right)}{2^{\left|A_{i}\right|}-1}\geq \;0\\ E\left(m_{2}\right)=-\sum_{i=1}^{N}m_{2}\left(A_{i}\right)\log_{2}\frac{m_{2}\left(A_{i}\right)}{2^{\left|A_{i}\right|}-1}\geq \;0\\ E_{m_{2}}\left(m_{1}\right)=-\sum_{i=1}^{N}m_{2}\left(A_{i}\right)\log_{2}\frac{m_{1}\left(A_{i}\right)}{2^{\left|A_{i}\right|}-1}\geq \;0\\ E_{m_{1}}\left(m_{2}\right)=-\sum_{i=1}^{N}m_{1}\left(A_{i}\right)\log_{2}\frac{m_{2}\left(A_{i}\right)}{2^{\left|A_{i}\right|}-1}\geq 0 \end{array}$$

Therefore,

$$\gamma \left(m_{1};m_{2}\right)=\frac{E\left(m_{1}\right)+E\left(m_{2}\right)}{E_{m_{1}}\left(m_{2}\right)+E_{m_{2}}\left(m_{1}\right)}\geq 0$$

Thus, the non-negativity of the belief correlation measure is proved. □

**Property** **3** (Non-degeneracy)**.** *$\gamma (m_{1};m_{2})=1$ if and only if $m_{1}=m_{2}$.*

**Proof.**  Firstly consider $m_{1}=m_{2}$, it can be obtained that:

$$E\left(m_{1}\right)=E\left(m_{2}\right)=E_{m_{2}}\left(m_{1}\right)=E_{m_{1}}\left(m_{2}\right)$$

Thus,

$$\gamma \left(m_{1};m_{2}\right)=\frac{E\left(m_{1}\right)+E\left(m_{2}\right)}{E_{m_{1}}\left(m_{2}\right)+E_{m_{2}}\left(m_{1}\right)}=1$$

Next, when $\gamma (m_{1};m_{2})=1$, that is:

$$\frac{E\left(m_{1}\right)+E\left(m_{2}\right)}{E_{m_{1}}\left(m_{2}\right)+E_{m_{2}}\left(m_{1}\right)}=1$$

It can be obtained that:

$$E\left(m_{1}\right)+E\left(m_{2}\right)=E_{m_{1}}\left(m_{2}\right)+E_{m_{2}}\left(m_{1}\right)$$

Through the derivation process:

$$\begin{array}{c} -\sum_{i=1}^{N}m_{1}\left(A_{i}\right)\log_{2}\frac{m_{1}\left(A_{i}\right)}{2^{\left|A_{i}\right|}-1}-\sum_{i=1}^{N}m_{2}\left(A_{i}\right)\log_{2}\frac{m_{2}\left(A_{i}\right)}{2^{\left|A_{i}\right|}-1}=-\sum_{i=1}^{N}m_{2}\left(A_{i}\right)\log_{2}\frac{m_{1}\left(A_{i}\right)}{2^{\left|A_{i}\right|}-1}-\sum_{i=1}^{N}m_{1}\left(A_{i}\right)\log_{2}\frac{m_{2}\left(A_{i}\right)}{2^{\left|A_{i}\right|}-1}\\ \Rightarrow \sum_{i=1}^{N}m_{1}\left(A_{i}\right)\log_{2}m_{1}\left(A_{i}\right)+\sum_{i=1}^{N}m_{2}\left(A_{i}\right)\log_{2}m_{2}\left(A_{i}\right)=\sum_{i=1}^{N}m_{2}\left(A_{i}\right)\log_{2}m_{1}\left(A_{i}\right)+\sum_{i=1}^{N}m_{1}\left(A_{i}\right)\log_{2}m_{2}\left(A_{i}\right)\\ \Rightarrow \sum_{i=1}^{N}\left(m_{1}\left(A_{i}\right)-m_{2}\left(A_{i}\right)\right)\log_{2}m_{1}\left(A_{i}\right)=\sum_{i=1}^{N}(m_{1}\left(A_{i}\right)-m_{2}\left(A_{i}\right))\log_{2}m_{2}\left(A_{i}\right)\\ \Rightarrow \sum_{i=1}^{N}\left(m_{1}\left(A_{i}\right)-m_{2}\left(A_{i}\right)\right)m_{1}\left(A_{i}\right)=\sum_{i=1}^{N}(m_{1}\left(A_{i}\right)-m_{2}\left(A_{i}\right))m_{2}\left(A_{i}\right) \end{array}$$

Here, we consider it from the idea of disproof. Since the case that $m_{1}\neq m_{2}$ does not exist, making the above equation hold, it can be obtained that $m_{1}=m_{2}$. Therefore, the nondegeneracy of the belief correlation measure is proved. □

**Property** **4** (Boundedness)**.** *$0\leq \gamma (m_{1};m_{2})\leq 1$.*

**Proof.**  From Property 2, it has been proved that $\gamma (m_{1};m_{2})\geq 0$. Next, we prove the upper bound of $\gamma (m_{1};m_{2})$.

Though the derivation process of Equation (16), it can be obtained that:

$$-\sum_{i=1}^{k}p_{i}\log_{2}\frac{q_{i}}{c}\geq -\sum_{i=1}^{k}p_{i}\log_{2}\frac{p_{i}}{c}\geq 0$$

Then, we have:

$$\begin{array}{c} -\sum_{i=1}^{N}m_{2}\left(A_{i}\right)\log_{2}\frac{m_{1}\left(A_{i}\right)}{2^{\left|A_{i}\right|}-1}\geq -\sum_{i=1}^{N}m_{1}\left(A_{i}\right)\log_{2}\frac{m_{1}\left(A_{i}\right)}{2^{\left|A_{i}\right|}-1}\geq \;0\\ -\sum_{i=1}^{N}m_{1}\left(A_{i}\right)\log_{2}\frac{m_{2}\left(A_{i}\right)}{2^{\left|A_{i}\right|}-1}\geq -\sum_{i=1}^{N}m_{2}\left(A_{i}\right)\log_{2}\frac{m_{2}\left(A_{i}\right)}{2^{\left|A_{i}\right|}-1}\geq 0 \end{array}$$

With Equations (6) and (12), it can be obtained that:

$$\begin{array}{c} E_{m_{2}}\left(m_{1}\right)\geq E\left(m_{1}\right)\geq \;0\\ E_{m_{1}}\left(m_{2}\right)\geq E\left(m_{2}\right)\geq 0 \end{array}$$

Therefore,

$$\gamma \left(m_{1};m_{2}\right)=\frac{E\left(m_{1}\right)+E\left(m_{2}\right)}{E_{m_{1}}\left(m_{2}\right)+E_{m_{2}}\left(m_{1}\right)}\leq 1.$$

Thus, the boundedness of the belief correlation measure $0\leq \gamma (m_{1};m_{2})\leq 1$ is proved. □

**Property** **5** (Orthogonality)**.** *$\gamma (m_{1};m_{2})=0$ if and only if $m_{1}$ and $m_{2}$ are orthogonal.*

**Proof.**  When $m_{1}$ and $m_{2}$ are orthogonal, it should satisfy that: $A_{i}\cap A_{j}=\phi$, where $A_{i}$ and $A_{j}$ are propositions in mass functions $m_{1}$ and $m_{2}$, respectively. From Equation (6), it can be obtained that:

$$E\left(m_{1}\right)=\left\{\begin{array}{cc} 0, & \exists \;m_{1}\left(A_{i}\right)=1\;and\;\;A_{i}\in \Theta \\ c_{1}, & others \end{array}\right.$$

$$E\left(m_{2}\right)=\left\{\begin{array}{cc} 0, & \exists \;m_{2}\left(A_{j}\right)=1\;and\;\;A_{j}\in \Theta \\ c_{2}, & others \end{array}\right.$$

where $c_{1}$ and $c_{2}$ are constants. Under the orthogonality condition, there exists $m_{1}\left(A_{i}\right)=0$ and $m_{2}\left(A_{j}\right)=0$. Then, from Equation (12), it can be obtained that:

$$\begin{array}{c} E_{m_{1}}\left(m_{2}\right)\to +\infty ,\\ E_{m_{2}}\left(m_{1}\right)\to +\infty \end{array}$$

Therefore,

$$\gamma \left(m_{1};m_{2}\right)=\frac{E\left(m_{1}\right)+E\left(m_{2}\right)}{E_{m_{1}}\left(m_{2}\right)+E_{m_{2}}\left(m_{1}\right)}=0$$

Then, the orthogonality of the belief correlation measure is proved. □

**Property** **6** (Symmetry)**.** *$\gamma \left(m_{1};m_{2}\right)=\gamma \left(m_{2};m_{1}\right)$.*

**Proof.**  According to Equation (15), it can be obtained that:

$$\gamma \left(m_{1};m_{2}\right)=\frac{E\left(m_{1}\right)+E\left(m_{2}\right)}{E_{m_{1}}\left(m_{2}\right)+E_{m_{2}}\left(m_{1}\right)}$$

$$\gamma \left(m_{2};m_{1}\right)=\frac{E\left(m_{2}\right)+E\left(m_{1}\right)}{E_{m_{2}}\left(m_{1}\right)+E_{m_{1}}\left(m_{2}\right)}$$

Therefore,

$$\gamma \left(m_{1};m_{2}\right)=\gamma \left(m_{2};m_{1}\right)$$

Finally, the symmetry of the belief correlation measure is proved. □

Through the above proof processes, it is verified that the proposed belief correlation measure has probabilistic consistency, non-negativity, non-degeneracy, orthogonality, boundedness, and symmetry. It should be indicated that the two properties, orthogonality and triangular inequality, are in conflict. The belief correlation measure satisfies orthogonality, so it does not satisfy the triangle inequality.

## 4. Numerical Examples

This part provides the following numerical examples to illustrate the advantages of the belief correlation measure, and also verify its validity by comparing with other correlation measures.

Firstly, Example 1 is described in steps to illustrate the calculation procedure of the belief correlation measure and the rationality of the method.

**Example** **1.** *Assume there are three mass functions $m_{1},m_{2},m_{3}$ on the FOD Θ={C,D,E}:*

$$\begin{array}{c} m_{1}:m_{1}\left(C\right)=0.5,m_{1}\left(D\right)=0.4,m_{1}\left(C,E\right)=0.1;\\ m_{2}:m_{2}\left(C\right)=0.6,m_{2}\left(D\right)=0.3,m_{2}\left(C,E\right)=0.1;\\ m_{3}:m_{3}\left(C\right)=0.8,m_{3}\left(D\right)=0.1,m_{3}\left(C,E\right)=0.1. \end{array}$$

*Firstly, the belief entropy of $m_{1},m_{2}$, and $m_{3}$ can be obtained by Equation (6):*

$$\begin{array}{c} E\left(m_{1}\right)=-\left(0.5\times \log_{2}0.5+0.4\times \log_{2}0.4+0.1\times \log_{2}\frac{0.1}{2^{2}-1}\right)=1.5195\\ E\left(m_{2}\right)=-\left(0.6\times \log_{2}0.6+0.3\times \log_{2}0.3+0.1\times \log_{2}\frac{0.1}{2^{2}-1}\right)=1.4540\\ E\left(m_{3}\right)=-\left(0.8\times \log_{2}0.8+0.1\times \log_{2}0.1+0.1\times \log_{2}\frac{0.1}{2^{2}-1}\right)=1.0804 \end{array}$$

*Meanwhile, the partial belief entropy of $m_{1},m_{2}$, and $m_{3}$ can be obtained by Equation (12):*

$$\begin{array}{c} E_{m_{2}}\left(m_{1}\right)=-\left(0.6\times \log_{2}0.5+0.3\times \log_{2}0.4+0.1\times \log_{2}\frac{0.1}{2^{2}-1}\right)=1.4873\\ E_{m_{1}}\left(m_{2}\right)=-\left(0.5\times \log_{2}0.6+0.4\times \log_{2}0.3+0.1\times \log_{2}\frac{0.1}{2^{2}-1}\right)=1.5540\\ E_{m_{3}}\left(m_{1}\right)=-\left(0.8\times \log_{2}0.5+0.1\times \log_{2}0.4+0.1\times \log_{2}\frac{0.1}{2^{2}-1}\right)=1.4229\\ E_{m_{1}}\left(m_{3}\right)=-\left(0.5\times \log_{2}0.8+0.4\times \log_{2}0.1+0.1\times \log_{2}\frac{0.1}{2^{2}-1}\right)=1.9804\\ E_{m_{3}}\left(m_{2}\right)=-\left(0.8\times \log_{2}0.6+0.1\times \log_{2}0.3+0.1\times \log_{2}\frac{0.1}{2^{2}-1}\right)=1.2540\\ E_{m_{2}}\left(m_{3}\right)=-\left(0.6\times \log_{2}0.8+0.3\times \log_{2}0.1+0.1\times \log_{2}\frac{0.1}{2^{2}-1}\right)=1.6804 \end{array}$$

*Then, the belief correlation measure of $m_{1},m_{2}$, and $m_{3}$ can be calculated by Equation (15):*

$$\begin{array}{c} \gamma \left(m_{1};m_{2}\right)=\frac{E\left(m_{1}\right)+E\left(m_{2}\right)}{E_{m_{2}}\left(m_{1}\right)+E_{m_{1}}\left(m_{2}\right)}=\frac{1.5195+1.4540}{1.4873+1.5540}=0.9777\\ \gamma \left(m_{1};m_{3}\right)=\frac{E\left(m_{1}\right)+E\left(m_{3}\right)}{E_{m_{3}}\left(m_{1}\right)+E_{m_{1}}\left(m_{3}\right)}=\frac{1.5195+1.0804}{1.4229+1.9804}=0.7639\\ \gamma \left(m_{2};m_{3}\right)=\frac{E\left(m_{2}\right)+E\left(m_{3}\right)}{E_{m_{3}}\left(m_{2}\right)+E_{m_{2}}\left(m_{3}\right)}=\frac{1.4540+1.0804}{1.2540+1.6804}=0.8637 \end{array}$$

*From the above example, it can be seen intuitively that $m_{3}$ is more inconsistent with other evidence, and $m_{2}$ is more reliable. In the results obtained using this method, the average correlation degree of evidence $m_{2}$ and other evidence is $\frac{0.9777+0.8637}{2}=0.9207$, which is the highest. Meanwhile, the average correlation degree between $m_{3}$ and other evidence is $\frac{0.7639+0.8637}{2}=0.8138$, which is the lowest. This is consistent with intuition, indicating that the method is reasonable.*

In the following example, we calculate the belief correlation measure between the evidence corresponding to varying focal elements and mass values, thus illustrating the reliability of the proposed method.

**Example** **2.** *Suppose Θ={O,P,Q,⋯,X}, let t be a variable and t∈[0,1]. N is a variable subset, as in Table 1, and its number of elements n changes from 1 to 10. There are two mass functions:*

$$\begin{array}{c} m_{1}:m_{1}\left(\left\{O,P,Q,R,S\right\}\right)=0.2,m_{1}\left(N\right)=0.8;\\ m_{2}:m_{2}\left(\left\{O\right\}\right)=t,m_{2}\left(N\right)=1-t. \end{array}$$

**Figure 1:**
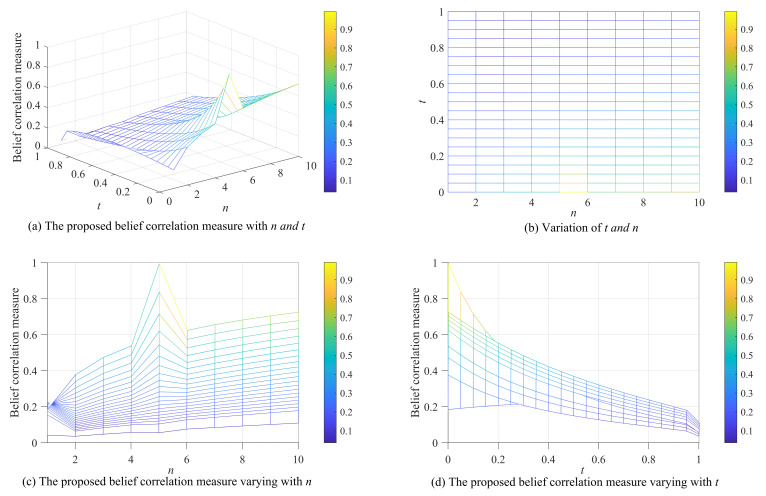
The calculation results of the belief correlation measure.

*The calculation results of the belief correlation measure are visualized as shown in Figure 1. Where the belief correlation measure changing with n and t is shown in Figure 1a. Figure 1b shows the variation intervals of variables n and t. Figure 1c shows the value of belief correlation measure varying with n. From this, we can see that when n=5, i.e., N={O,P,Q,R,S}, $m_{1}$ and $m_{2}$ have the highest consistency at this time, so the value of belief correlation measure at n=5 is much larger than other variable subsets. In more detail, since the distribution of focal elements in the two mass functions is different when n=1 and n=2, the belief correlation measure show different trend with the change of t. In other cases of n, the belief correlation measure generally tends to increase as n increases. This is because the correlation between the two mass functions increases as the subset N expands. Figure 1d shows the value of the belief correlation measure varying with t. From this, we can see that with the increase of t, the belief correlation measure generally shows a downward trend. This is because as t increases, the degree of variation between belief functions also increases, resulting in a decrease in correlation; this is consistent with our intuition.*

*Additionally, the values of the belief correlation measure all ranged from 0∼1, verifying that the belief correlation measure is bounded.*

In addition, for demonstrating the validity of this method, it is compared with other correlation measures, such as Jousselme’s Distance $d_{J}$  [57], Belief Jensen–Shannon divergence BJS [48], Plausibility and Belief Jensen–Shannon divergence PBl_BJS [58], and the correlation coefficient $r_{BPA}$ [46]. Since $d_{J}$, BJS, and PBl_BJS describe the evidence relationships through the differences between them, here we use $1-d_{J}$, 1−BJS, 1−PBl_BJS to measure the correlation between evidence for comparison as follows:

**Example** **3.** *Assume the FOD is Θ={C,D}, t is a value in the set {0, 0.1, 0.2, 0.3, …, 1}, two pieces of evidence are defined as:*

$$\begin{array}{c} m_{1}:m_{1}\left(C\right)=0.3,m_{1}\left(D\right)=0.4,m_{1}\left(C,D\right)=0.3\\ m_{2}:m_{2}\left(C\right)=0.5*\left(1-t\right),m_{2}\left(D\right)=t,m_{2}\left(C,D\right)=0.5*\left(1-t\right) \end{array}$$

**Figure 2:**
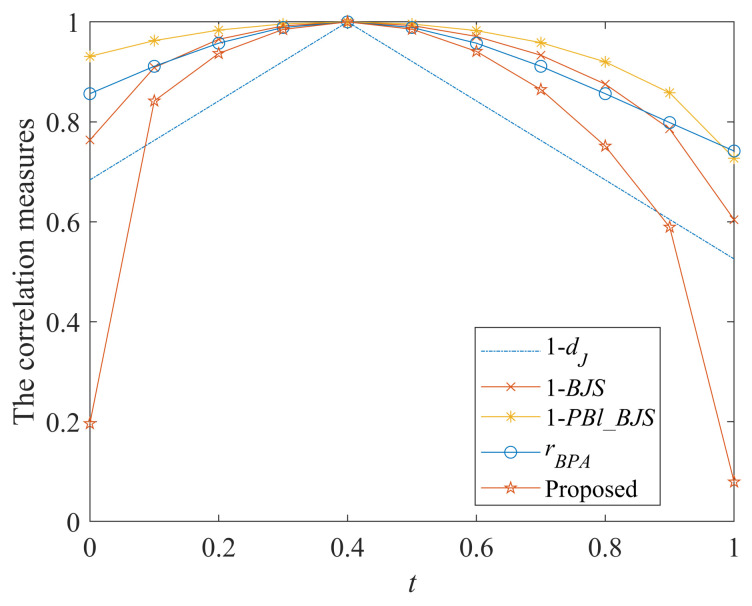
Comparisons of the correlation measures.

*Then, we obtain 11 pairs of BPAs and the comparisons of their correlation measures in Table 2 and Figure 2.*

*Table 2 and Figure 2 show that with the change of t, the change trend of these correlation measurement methods are consistent, and when t=0.4, i.e., $m_{1}$ and $m_{2}$ are exactly the same, the correlation degree value of all measurement methods are 1; this is reasonable. Since the mass values of $m_{2}$ change in the focal elements {C} and {C,D}, it is more reasonable that the result of the correlation measure is nonlinear.*

*From the details, when t=0, evidence $m_{2}$ is: $m_{2}\left(C\right)=0.5,m_{2}\left(D\right)=0,m_{2}\left(C,D\right)=0.5$, indicating that the evidence $m_{2}$ is completely unsupported for the focal element {D}, while the evidence $m_{1}$ has the highest support degree for {D}. At this time, the correlation between these two pieces of evidence should be small. In addition, when t=1, evidence $m_{2}$ is: $m_{2}\left(C\right)=0,$$m_{2}\left(D\right)=1,$$m_{2}\left(C,D\right)=0$, indicating that evidence $m_{2}$ fully supports {D}, and does not support {C} and {C,D} at all, and the evidence $m_{1}$ has a similar support degree for each focal element. At this time, the correlation between them should also be smaller. It is unreasonable that other comparison correlation measures maintain relatively high correlation values for evidence $m_{2}$ at both t=0 and t=1. However, the proposed belief correlation measure has low correlation values at both t=0 and t=1, indicating that the method has a high sensitivity in terms of uncertainty. This is because this method integrates belief entropy, which makes it possible to calculate the correlation of belief functions by also taking uncertainty into account, thus making the calculation more reasonable.*

## 5. A New Information Fusion Method Based on Belief Correlation Measure

Based on the belief correlation measure, this paper proposes an information fusion method for multi-source data. This method considers not only the correlation between belief functions, but also their own discriminability to introduce objective weight and subjective weight, respectively. Specifically, it utilizes the belief correlation measure to quantify the support degree between belief functions for generating objective weights. Meanwhile, the discriminability measure is used to quantify the certainty of the belief function to generate subjective weights. Then, the combinational weight is designed to perform evidence fusion, which can improve the rationality and reliability of decision results.

**Figure 3:**
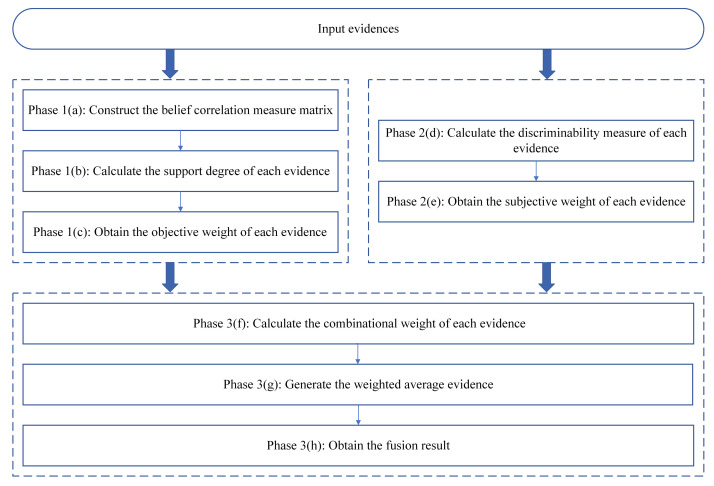
Process diagram of the proposed information fusion method.

This part first introduces the information fusion method in phases, especially the weights of belief functions that need to be determined in the process. Figure 3 shows the process diagram of this method. Then, the procedures are described through an algorithm for readers to better understand.

Phase 1: Generate objective weights for each evidence.

(a) Assume that $m_{1},m_{2},\ldots ,m_{n}$ are *n* belief functions with independent sources on FOD $\Theta =\{\theta_{1},\theta_{2},\ldots ,\theta_{N}\}$. According to the proposed belief correlation measure, the belief correlation between $m_{i}$ and $m_{j}$ can be calculated by Equation (12)–(15). Then, the belief correlation measure matrix $BCMM={\left[\gamma \left(m_{i};m_{j}\right)\right]}_{n\times n}$ is constructed as follows:
  (17) $$BCMM=\left[\begin{array}{ccccc} 1 & \cdots & \gamma (m_{1};m_{j}) & \cdots & \gamma (m_{1};m_{n})\\ \vdots & \vdots & \vdots & \vdots & \vdots \\ \gamma (m_{i};m_{1}) & \cdots & 1 & \cdots & \gamma (m_{i};m_{n})\\ \vdots & \vdots & \vdots & \vdots & \vdots \\ \gamma (m_{n};m_{1}) & \cdots & \gamma (m_{n};m_{j}) & \cdots & 1 \end{array}\right]$$
(b) According to the belief correlation measure matrix BCMM, the support degree of $m_{i}$ is defined as:
  (18) $$Sup\left(m_{i}\right)=\sum_{j=1,j\neq i}^{n}\gamma (m_{i},m_{j})$$
(c) On the basis of $Sup(m_{i})$, the objective weight of $m_{i}$ can be generated:
  (19) $$w^{O}\left(m_{i}\right)=\frac{Sup(m_{i})}{\sum_{i=1}^{n}Sup(m_{i})}$$
  The objective weight can reflect the credibility of evidence to some extent. A larger value of $w^{O}\left(m_{i}\right)$ indicates that the evidence $m_{i}$ is supported by other evidence to a greater extent, indicating that its credibility is higher.

Phase 2: Generate subjective weight for each evidence.

(d) The subjective weight can be analyzed based on the certainty of evidence itself, which can be obtained by calculating the discriminability measure (DM):
  (20) $$DM\left(m_{i}\right)=\max_{\theta \in \Theta }\left(BetP_{m_{i}}\left(\theta \right)\right)-\max_{\theta_{k}\in \Theta ,\theta \neq \theta_{k}}\left(BetP_{m_{i}}\left(\theta_{k}\right)\right)$$
  where $BetP_{m}$ is the pignistic probability transformation [59]:
  (21) $$BetP_{m}\left(\theta \right)=\sum_{\theta \in B\subseteq \Theta }\frac{1}{\left|B\right|}\frac{m(B)}{1-m(\phi )}$$
  According to the belief functions $m_{1},m_{2},\ldots ,m_{n}$, the discriminability measure of each evidence can be calculated and denoted as $DM(m_{i})$.
(e) Then, the subjective weight of evidence can be calculated:
  (22) $$w^{S}\left(m_{i}\right)=\frac{DM(m_{i})}{\sum_{i=1}^{n}DM(m_{i})}$$
  The subjective weight can reflect the certainty of evidence. A larger value of $w^{S}\left(m_{i}\right)$ indicates that more certainty information is provided by evidence $m_{i}$; then, it has a higher usability.

Phase 3: Generate new evidence and perform information fusion.

(f) According to the objective weight $w^{O}\left(m_{i}\right)$ obtained by Step 1 and the subjective weight $w^{S}\left(m_{i}\right)$ obtained by Step 2, the combinational weight is defined as:
  (23) $$w^{C}\left(m_{i}\right)=\lambda w^{O}\left(m_{i}\right)+\left(1-\lambda \right)w^{S}\left(m_{i}\right)$$
  where λ is an adjusting coefficient that takes a value between 0 and 1. It represents the relative importance of the subjective weight and objective weight. Under normal circumstances, the objective and subjective weight are considered equally important, the value of λ is 0.5.
(g) After determining the combinational weight $w^{C}\left(m_{i}\right)$, a new evidence $\tilde{m}\left(\theta \right)$ is generated by the weighted average operation:
  (24) $$\tilde{m}\left(\theta \right)=\sum_{i=1}^{n}w^{C}\left(m_{i}\right)\times m_{i}\left(\theta \right),\theta \in \Theta$$
(h) The generated evidence is combined for (n−1) times according to Dempster’s combination rule to obtain the fusion result:
  (25) $${\tilde{m}}_{F}=\underset{(n-1)times}{\underset{︸}{\tilde{m}⊕\tilde{m}⊕\cdots ⊕\tilde{m}}}.$$

In the proposed information fusion method, the belief correlation measure and discriminability measure are utilized to generate the objective and subjective weights. The method takes into account the relational information, as well as the certainty information, to determine the combinational weight, which can reflect the credibility and usability of evidence in an integrated manner. For readers to understand the method better, the corresponding pseudo-code is given to illustrate the combination process in Algorithm 1.
**Algorithm 1:** An algorithm for the proposed information fusion method
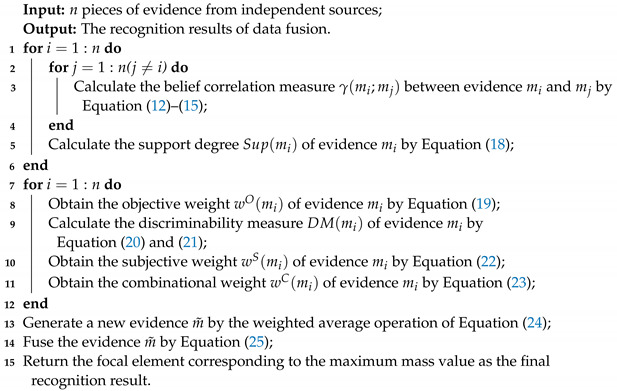


## 6. Application in Multi-Source Data Fusion

This part, the application in the field of multi-source data fusion, is studied to validate the proposed information fusion method, which is based on the belief correlation measurement. Since target recognition is a typical task that requires multi-sensor data fusion, two application cases for target recognition are given below.

Case 1: There is a target recognition task from Ref. [48], which acquires multi-source information based on different types of sensors. These sensors report the target type information as shown in Table 3. In this case, there are five different types of sensors $S_{i}$(i=1,2,3,4,5), and their corresponding BPAs are $m_{i}$. According to the sensor reports, there are three kinds that the target may be recognized as, which form the FOD $\Theta =\{A_{1},A_{2},A_{3}\}$.

**Figure 4:**
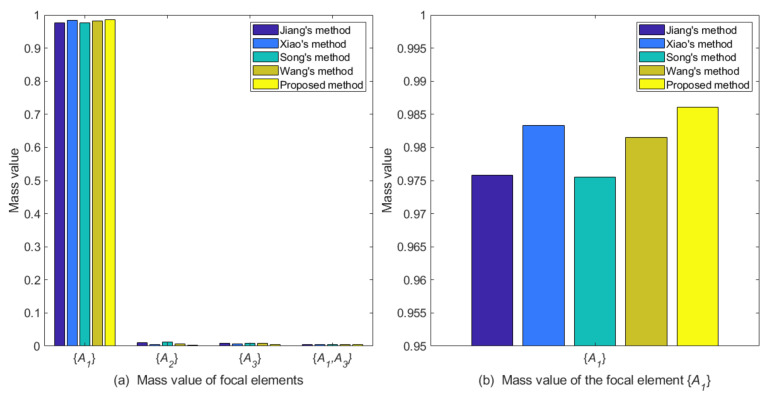
The comparation of different methods for Case 1.
  By analyzing the original evidence obtained by the sensors, it can be found that the BPA value reported by sensor $S_{1}$ supports the three targets $A_{1},A_{2},A_{3}$ to a similar degree, so the evidence is poor in usability. Furthermore, the BPA value reported by sensor $S_{2}$ has a large conflict with other evidence, indicating that the sensor $S_{2}$ may be abnormal or fault, so the evidence is unreliable. The method proposed in this paper assigns lower weight values to evidence $m_{1}$ and $m_{2}$, which is reasonable. As shown in Table 4 and Figure 4, the target recognition result of the proposed method is $A_{1}$, it is consistent with the recognition results of comparison methods. Meanwhile, the proposed method supports $A_{1}$ with a degree of 0.9861, which is higher than other comparison methods, and the decision result is more certain.
  Then, we conduct a sensitivity analysis on the values of parameter λ. When λ takes a different value between 0 and 1, the change trends of the support degree to the target $A_{1}$ of different methods are shown in Figure 5. In this case, as the value of parameter λ increases, different methods have improved the support degree for target $A_{1}$, indicating that the objective weight has a positive impact on the support degree for target $A_{1}$, and the subjective weight has a negative impact on it. Moreover, the proposed method has a consistently higher support degree than other comparison methods.
  **Figure 5:**
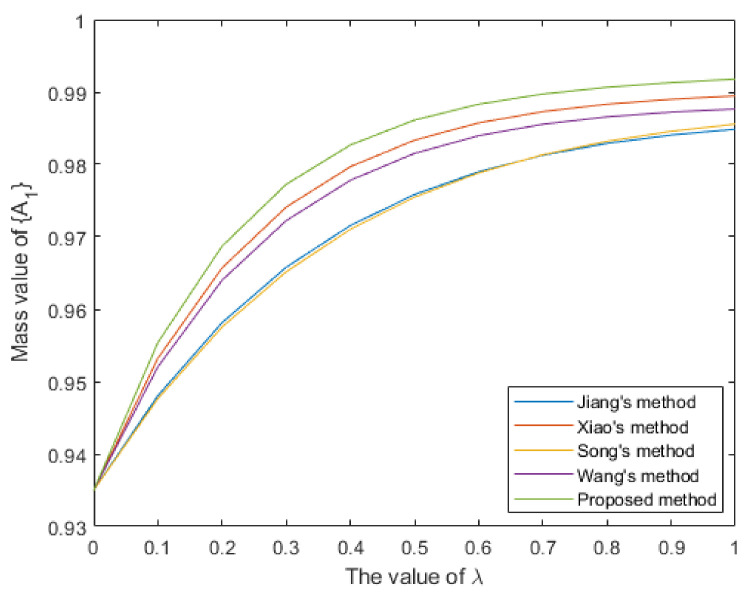
The sensitivity analysis on parameter λ for Case 1.
  To further demonstrate the validity of the proposed belief correlation measure, we perform a statistical test by adding different levels of noise to evidence and analyze the fusion results when λ takes the value of 1. The sensor evidence with the highest support degree to target is selected to add noise. The noise makes the sensor evidence decrease the support degree to target and increase the unknown. Specifically, the focal element with the largest mass value in the original evidence is selected, that is the focal element $\left\{A_{1}\right\}$ in $m_{5}$. Then, its mass value is reduced and the reduced part of the mass value is assigned to the Θ of the corresponding evidence. After adding noise, the focal elements in $m_{5}$ become:
  $$m_{5}^{\prime }:m_{5}^{\prime }\left\{A_{1}\right\}=0.6-v,m_{5}^{\prime }\left\{A_{2}\right\}=0.1,m_{5}^{\prime }\left\{A_{1},A_{3}\right\}=0.3,m_{5}^{\prime }\left\{\Theta \right\}=v$$
  where *v* denotes the variation of the mass value due to the addition of noise. Then data fusion is conducted based on different correlation measurement methods. Table 5 records the mass values of focal element $\left\{A_{1}\right\}$ in the fusion results under different variation values and their average is statistically calculated. The visualization is shown in Figure 6. From the experimental results it can be seen that the proposed method always has a higher support for focal element $\left\{A_{1}\right\}$ under different levels of noise conditions. The statistical average values show that there is a significant difference between the proposed method and the comparison methods. It is verified that the proposed method is more beneficial for decision making.
  **Table 5:** The mass values of focal element $\left\{A_{1}\right\}$ in fusion results under different variation values.

- Implementation by the proposed method
  Step 1: Generate objective weights for each evidence.
  (a) The belief correlation measure matrix $BCMM={\left(\gamma_{ij}\right)}_{5\times 5}$ can be constructed as:
    $$BCMM=\left[\begin{array}{ccccc} 1 & 0.1062 & 0.1173 & 0.1206 & 0.1254\\ 0.1062 & 1 & 0.0505 & 0.0544 & 0.0517\\ 0.1173 & 0.0505 & 1 & 0.9952 & 0.9924\\ 0.1206 & 0.0544 & 0.9952 & 1 & 0.9953\\ 0.1254 & 0.0517 & 0.9924 & 0.9953 & 1 \end{array}\right]$$
  (b) According to the belief correlation measure matrix BCMM, the $Sup(m_{i})$ are calculated:
    $$\begin{array}{c} Sup(m_{1})=0.4696\\ Sup(m_{2})=0.2628\\ Sup(m_{3})=2.1555\\ Sup(m_{4})=2.1655\\ Sup(m_{5})=2.1647 \end{array}$$
  (c) Then, the objective weight $w^{O}\left(m_{i}\right)$ of each evidence can be obtained:
    $$\begin{array}{c} w^{O}\left(m_{1}\right)=0.0651\\ w^{O}\left(m_{2}\right)=0.0364\\ w^{O}\left(m_{3}\right)=0.2986\\ w^{O}\left(m_{4}\right)=0.3000\\ w^{O}\left(m_{5}\right)=0.2999 \end{array}$$
    Step 2: Generate subjective weight for each evidence.
  (d) According to the BPA value of each, the discriminability measures $DM(m_{i})$ are calculated as:
    $$\begin{array}{c} DM(m_{1})=0.1100\\ DM(m_{2})=0.8000\\ DM(m_{3})=0.5800\\ DM(m_{4})=0.5500\\ DM(m_{5})=0.6000 \end{array}$$
  (e) Based on the discriminability measure, the subjective weight $w^{S}\left(m_{i}\right)$ of each evidence value can be obtained as:
    $$\begin{array}{c} w^{S}\left(m_{1}\right)=0.0417\\ w^{S}\left(m_{2}\right)=0.3030\\ w^{S}\left(m_{3}\right)=0.2197\\ w^{S}\left(m_{4}\right)=0.2083\\ w^{S}\left(m_{5}\right)=0.2273 \end{array}$$
    Step 3: Generate new evidence and perform information fusion.
  (f) Based on the objective weight obtained by Step 1 and the subjective weight obtained by Step 2, and set the value of λ to 0.5, the combinational weight $w^{C}\left(m_{i}\right)$ can be calculated as:
    $$\begin{array}{c} w^{C}\left(m_{1}\right)=0.0534\\ w^{C}\left(m_{2}\right)=0.1697\\ w^{C}\left(m_{3}\right)=0.2592\\ w^{C}\left(m_{4}\right)=0.2542\\ w^{C}\left(m_{5}\right)=0.2636 \end{array}$$
  (g) The new evidence generated by the weighted average operation is obtained as:
    $$\begin{array}{c} \tilde{m}\left(\left\{A_{1}\right\}\right)=0.4701\\ \tilde{m}\left(\left\{A_{2}\right\}\right)=0.2381\\ \tilde{m}\left(\left\{A_{3}\right\}\right)=0.0330\\ \tilde{m}\left(\left\{A_{1},A_{3}\right\}\right)=0.2588 \end{array}$$
  (h) The generated evidence is combined four times to obtain the fusion results:
    $$\begin{array}{c} {\tilde{m}}_{F}\left(\left\{A_{1}\right\}\right)=0.9861\\ {\tilde{m}}_{F}\left(\left\{A_{2}\right\}\right)=0.0037\\ {\tilde{m}}_{F}\left(\left\{A_{3}\right\}\right)=0.0046\\ {\tilde{m}}_{F}\left(\left\{A_{1},A_{3}\right\}\right)=0.0056 \end{array}$$
- Comparison and discussion
  The proposed belief correlation measure is compared with other correlation measurement methods. Among the contrast methods, Xiao’s method [48], Song’s method [56], and Wang’s method [58] use the reciprocal of the divergence measure they proposed to represent the correlation between evidence. In order to conduct a comparative analysis more objectively, the correlation of each evidence obtained by other correlation measurement methods are normalized as objective weights, and the discriminability measure is used as the subjective weight. Next, the combinational weight is used to generate the weighted average evidence. Figure 4 and Table 4 display the experimental results, where the value of parameter λ in all methods is 0.5.
  **Table 4:** Fusion results of comparison methods in Case 1.

Method	$\left\{A_{1}\right\}$	$\left\{A_{2}\right\}$	$\left\{A_{3}\right\}$	$\left\{A_{1},A_{3}\right\}$	Target
Jiang’s method [46]	0.9758	0.0106	0.0094	0.0041	$A_{1}$
Xiao’s method [48]	0.9833	0.0050	0.0069	0.0048	$A_{1}$
Song’s method [56]	0.9755	0.0116	0.0084	0.0045	$A_{1}$
Wang’s method [58]	0.9815	0.0058	0.0084	0.0043	$A_{1}$
Proposed method	0.9861	0.0037	0.0046	0.0056	$A_{1}$

Variation	Jiang’s Method	Xiao’s Method	Song’s Method	Wang’s Method	Proposed
0.30	0.9241	0.9242	0.9207	0.9102	0.9548
0.32	0.9198	0.9165	0.9158	0.9000	0.9528
0.34	0.9156	0.9083	0.9109	0.8890	0.9509
0.36	0.9114	0.8997	0.9059	0.8772	0.9492
0.38	0.9072	0.8906	0.9008	0.8646	0.9475
0.40	0.9031	0.8812	0.8957	0.8514	0.9459
0.42	0.8991	0.8715	0.8906	0.8376	0.9445
0.44	0.8952	0.8617	0.8855	0.8233	0.9433
0.46	0.8915	0.8519	0.8804	0.8087	0.9422
0.48	0.8880	0.8422	0.8754	0.7938	0.9412
0.50	0.8847	0.8328	0.8705	0.7789	0.9405
Average	0.9036	0.8801	0.8957	0.8486	0.9466

**Figure 6:**
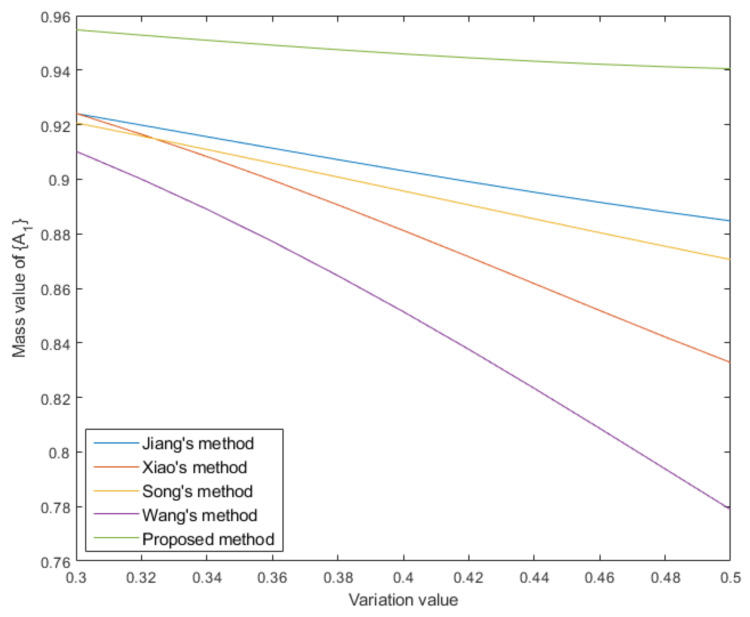
Visualization of mass value of focal element $\left\{A_{1}\right\}$ under different variation values.

Case 2: In another target recognition task [56], the possible targets constitute the frame of discernment $\Omega =\{B_{1},B_{2},B_{3}\}$. There are four sensors in the sensor system that report the target type information in Table 6.

**Figure 7:**
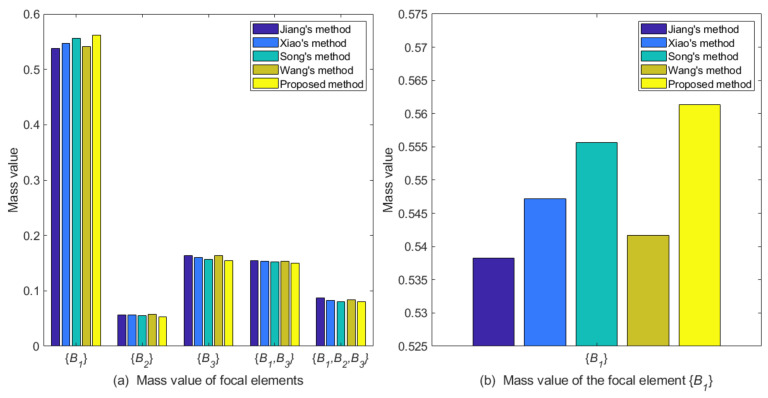
The comparison of different methods for Case 2.

Since the implementation process of this case is consistent with Case 1, it will not be repeated in this part. When compared with other correlation measurement methods, the normalized correlation degree calculated by each method is still used as the objective weight, and the discriminability measure is used as the subjective weight. Table 7 and Figure 7 display the fusion results.

By analyzing the original evidence obtained by the sensors, it can be seen that the BPA value of sensor $S_{1}$ for $B_{1}$ was 0, which is in great conflict with other evidence. The BPA value of the focal element $\left\{B_{1},B_{2},B_{3}\right\}$ was 0.7, indicating that the evidence has uncertainty to a large extent. Therefore, the proposed method gives evidence $m_{1}$ a lower weight. Sensor $S_{2}$ and sensor $S_{4}$ are more inclined to support target $B_{1}$, and the evidence they correspond to has higher certainty and less conflict with other evidence, so it is reasonable that they are given higher weights. From Table 7 and Figure 7, it can be seen that the recognition result of the proposed method is consistent with other comparison methods, and supports $B_{1}$ with a degree of 0.5614, which is higher than other comparison methods. This shows that the proposed method of the belief correlation measure is more effective.

**Figure 8:**
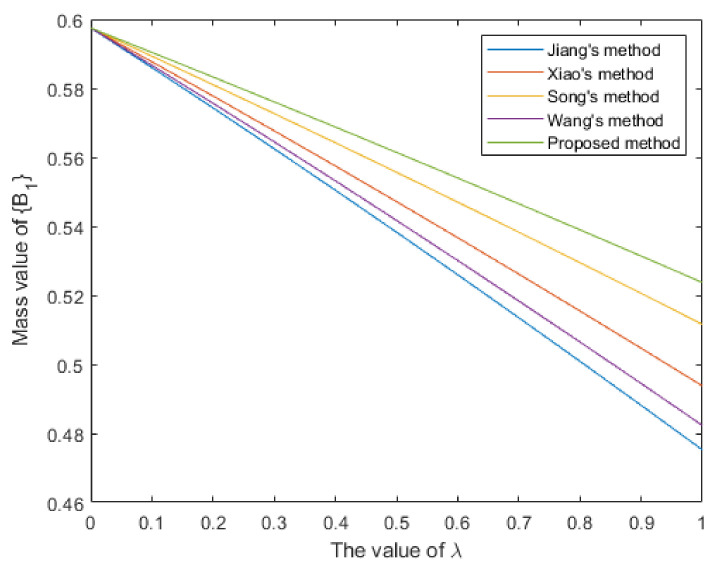
The sensitivity analysis on parameter λ for Case 2.

We also conducted a sensitivity analysis of each method on the value of parameter λ. When λ takes a different value between 0 and 1, the change trends of the support degree to the target $B_{1}$ of different methods are shown in Figure 8. It can be found that as the parameter λ increases, the fusion results of different methods have reduced the support degree for target $B_{1}$, indicating that, in this case, the objective weight had a negative impact on the support degree for target $B_{1}$, and the subjective weight had a positive impact on it. The proposed method always had a higher support degree than other comparison methods.

Then, a statistical test by adding different levels of noise to evidence was also performed. Specifically, the focal element $\left\{B_{1}\right\}$ in $m_{4}$ was selected to add noise. After adding noise, the focal elements in $m_{4}$ became:

$$m_{4}^{\prime }:m_{4}^{\prime }\left\{B_{1}\right\}=0.3-v,m_{4}^{\prime }\left\{B_{2}\right\}=0.05,m_{4}^{\prime }\left\{B_{3}\right\}=0.1,m_{4}^{\prime }\left\{B_{1},B_{3}\right\}=0.1,m_{4}^{\prime }\left\{\Theta \right\}=0.45+v$$

**Figure 9:**
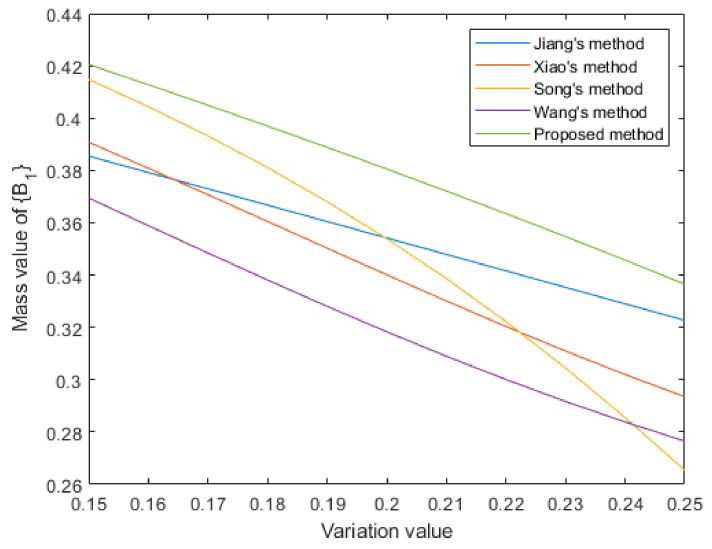
The visualization for mass value of focal element $\left\{B_{1}\right\}$ under different variation values.

Then, data fusion was conducted based on different correlation measurement methods. Table 8 and Figure 9 display the experimental results. It can be seen that when the variation value was small, the support degree of Song’s method was similar with the proposed method. However, when the variation value became larger, its support for $\left\{B_{1}\right\}$ decreased dramatically. The proposed method always had the highest support for $\left\{B_{1}\right\}$ under different levels of noise conditions. The statistical average value of the proposed method was higher than other comparison methods. It is verified that the proposed method is effective.

## 7. Conclusions

In this work, a new correlation measure for belief functions is proposed on the basis of belief entropy and relative entropy. The proposed belief correlation measure takes into account the influence of information uncertainty on the relevance between belief functions. In addition, this measure has some important mathematical properties of probabilistic consistency, non-negativity, non-degeneracy, boundedness, orthogonality, and symmetry. Based on the belief correlation measure and discriminability measure, a new information fusion method is designed. This fusion method uses the belief correlation measure between evidence to generate the objective weight, and uses the discriminability measure of evidence to generate the subjective weight. Then, the combinational weight is obtained, which can reflect more comprehensive information of evidence. The information fusion method can be applied to multi-source data processing tasks. Two specific application cases are described in steps to demonstrate the reliability of the proposed method. Through comparison and analysis with existing methods, it is verified that the proposed method is effective.

It should be noted that the proposed belief correlation measure satisfies the property of orthogonality but does not satisfy triangular inequality, which conflicts with orthogonality. We will conduct further work to improve the consistency of this approach. In addition, we also tend to integrate the information fusion method into recognition or control systems to construct end-to-end models in future studies, which may further enhance the performance of information processing systems.

## Figures and Tables

**Table 1:** The variable subset *N*.

*n*	*N*
1	{O}
2	{O,P}
3	{O,P,Q}
4	{O,P,Q,R}
5	{O,P,Q,R,S}
6	{O,P,Q,R,S,T}
7	{O,P,Q,R,S,T,U}
8	{O,P,Q,R,S,T,U,V}
9	{O,P,Q,R,S,T,U,V,W}
10	{O,P,Q,R,S,T,U,V,W,X}

**Table 2:** Comparisons of the correlation measures in Example 3.

t	$1-d_{\mathrm{BPA}}$	1−BJS	1−PBl_BJS	$r_{\mathrm{BPA}}$	Proposed
0	0.6838	0.7635	0.9311	0.8563	0.1964
0.1	0.7628	0.9087	0.9626	0.9111	0.8419
0.2	0.8419	0.9651	0.9837	0.9574	0.9363
0.3	0.9209	0.9921	0.9959	0.9888	0.985
0.4	1	1	1	1	1
0.5	0.9209	0.9927	0.9958	0.9888	0.9854
0.6	0.8419	0.971	0.9826	0.9574	0.941
0.7	0.7628	0.9333	0.9586	0.9111	0.8647
0.8	0.6838	0.8755	0.92	0.8563	0.7515
0.9	0.6047	0.7859	0.858	0.7985	0.5894
1	0.5257	0.6042	0.7272	0.7416	0.0796

**Table 3:** The BPAs of sensor reports in Case 1.

	$\left\{A_{1}\right\}$	$\left\{A_{2}\right\}$	$\left\{A_{3}\right\}$	$\left\{A_{1},A_{3}\right\}$
$S_{1}:m_{1}\left(\cdot \right)$	0.41	0.29	0.3	0
$S_{2}:m_{2}\left(\cdot \right)$	0	0.9	0.1	0
$S_{3}:m_{3}\left(\cdot \right)$	0.58	0.07	0	0.35
$S_{4}:m_{4}\left(\cdot \right)$	0.55	0.1	0	0.35
$S_{5}:m_{5}\left(\cdot \right)$	0.6	0.1	0	0.3

**Table 6:** The BPAs of sensor reports in Case 2.

	$\left\{B_{1}\right\}$	$\left\{B_{2}\right\}$	$\left\{B_{3}\right\}$	$\left\{B_{1},B_{3}\right\}$	$\left\{B_{1},B_{2},B_{3}\right\}$
$S_{1}:m_{1}\left(\cdot \right)$	0	0.08	0.12	0.1	0.7
$S_{2}:m_{2}\left(\cdot \right)$	0.3	0.05	0.05	0.2	0.4
$S_{3}:m_{3}\left(\cdot \right)$	0.15	0.1	0.1	0.15	0.5
$S_{4}:m_{4}\left(\cdot \right)$	0.3	0.05	0.1	0.1	0.45

**Table 7:** Fusion results of comparison methods in Case 2.

Method	$\left\{B_{1}\right\}$	$\left\{B_{2}\right\}$	$\left\{B_{3}\right\}$	$\left\{B_{1},B_{3}\right\}$	$\left\{B_{1},B_{2},B_{3}\right\}$	Target
Jiang’s method	0.5383	0.0559	0.1635	0.1547	0.0877	$B_{1}$
Xiao’s method	0.5472	0.0565	0.1608	0.1530	0.0825	$B_{1}$
Song’s method	0.5557	0.0552	0.1573	0.1520	0.0799	$B_{1}$
Wang’s method	0.5417	0.0576	0.1635	0.1530	0.0843	$B_{1}$
Proposed method	0.5614	0.0525	0.1551	0.1503	0.0807	$B_{1}$

**Table 8:** The mass values of focal element $\left\{B_{1}\right\}$ in fusion results under different variation values.

Variation	Jiang’s Method	Xiao’s Method	Song’s Method	Wang’s Method	Proposed
0.15	0.3854	0.3908	0.4148	0.3694	0.4205
0.16	0.3792	0.3808	0.4044	0.3589	0.4128
0.17	0.3730	0.3707	0.3932	0.3484	0.4049
0.18	0.3667	0.3605	0.3811	0.3381	0.3969
0.19	0.3605	0.3503	0.3680	0.3280	0.3888
0.20	0.3542	0.3401	0.3539	0.3183	0.3805
0.21	0.3479	0.3301	0.3387	0.3090	0.3721
0.22	0.3416	0.3203	0.3222	0.3001	0.3635
0.23	0.3353	0.3109	0.3044	0.2917	0.3547
0.24	0.3290	0.3020	0.2855	0.2838	0.3457
0.25	0.3227	0.2935	0.2654	0.2765	0.3365
Average	0.3541	0.3409	0.3483	0.3202	0.3797

## Data Availability

Data is contained within the article.
